# Supplementary material for: The momentum of the solar energy transition
Source: Nat Commun. 2023 Oct 17;14:6542. doi: 10.1038/s41467-023-41971-7 (PMC10582067; doi:10.1038/s41467-023-41971-7)
Supplement: Supplementary file 1 — Supplementary Information [file 41467_2023_41971_MOESM1_ESM.pdf]

Supplementary Material for

Is a solar future inevitable?

Femke J.M.M. Nijsse[1*], Jean-Francois Mercure[1,2,3], Nadia Ameli[4], Francesca Larosa[4,5], Sumit Kothari[4], Jamie Rickman[4], Pim Vercoulen[1,6], Hector Pollitt[2,3]

[1] Global Systems Institute, Department of Geography, University of Exeter, UK

[2] Cambridge Centre for Energy, Environment and Natural Resource Governance, University of Cambridge, UK.

[3] The World Bank, US

[4] Institute for Sustainable Resources, University College London, UK

[5] Royal Institute of Technology (KTH), Climate Action Centre, Sweden

[6] Cambridge Econometrics, Cambridge, UK

[*] email: f.j.m.m.nijsse@exeter.ac.uk

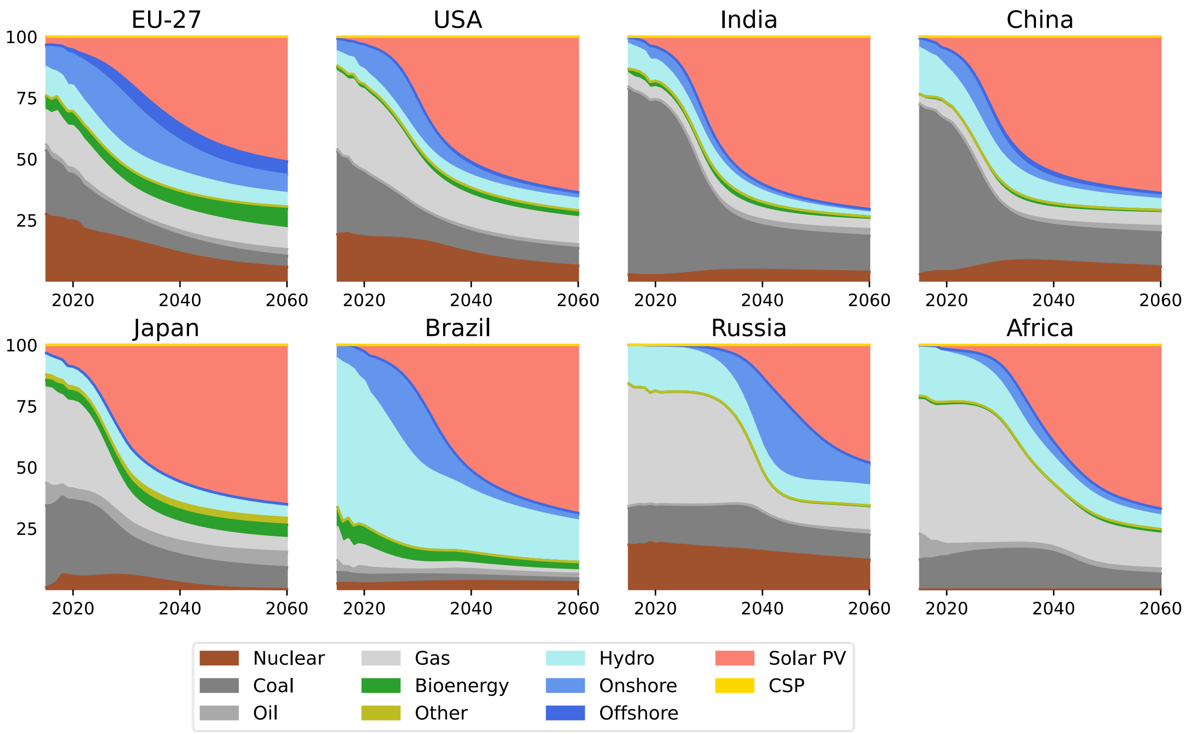

Supplementary Figure 1: ***Share of energy generation by region****. The same as Figure 1 of the main manuscript, grouped by region.*

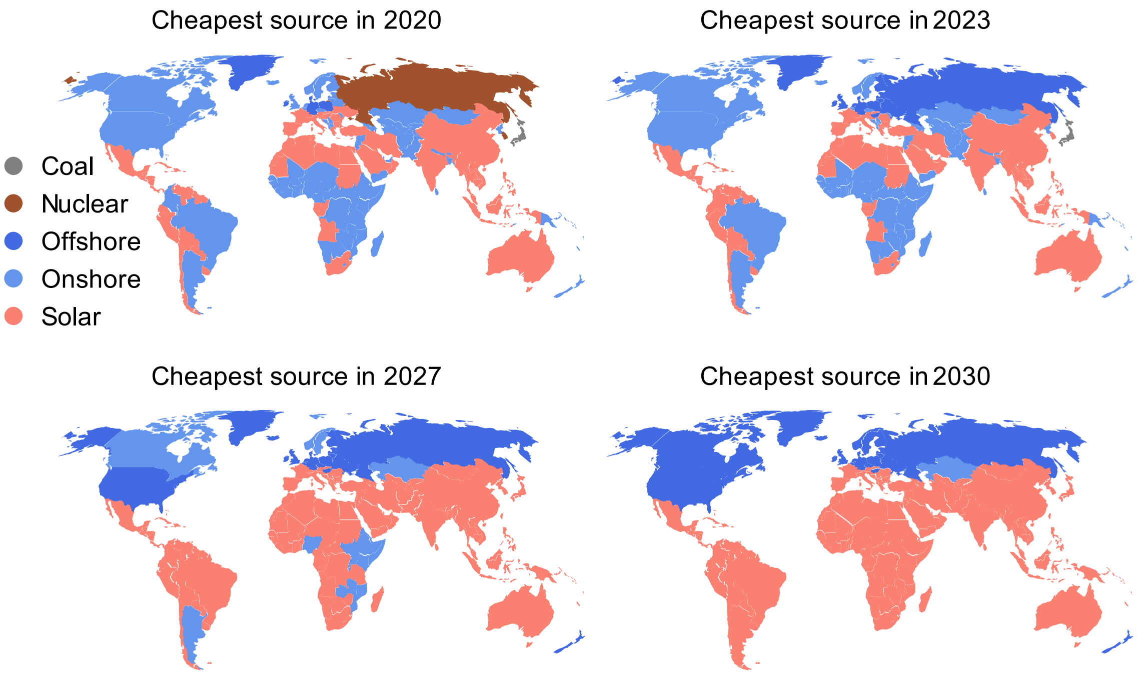

Supplementary Figure 2: the technology with the lowest system LCOE for the simulation runs with a very low share of solar power generation in 2050 (lowest 5% shares solar PV)

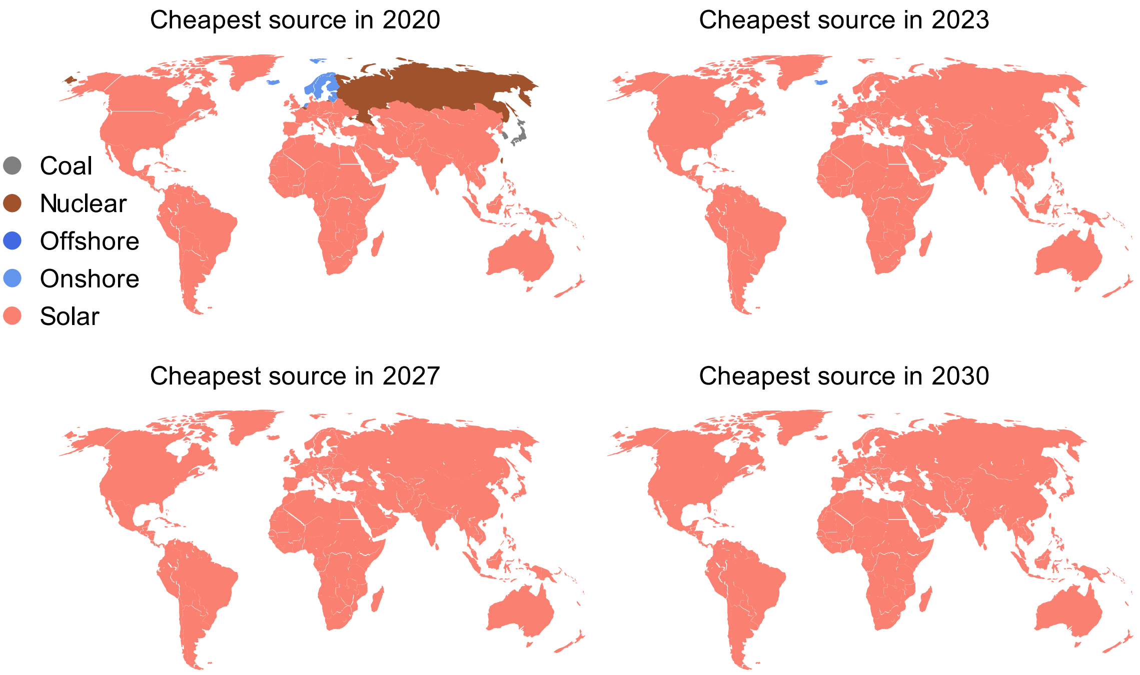

Supplementary Figure 3: the technology with the lowest system LCOE by region for the simulation runs with a very high share of solar power generation in 2050 (highest 5% shares solar PV)

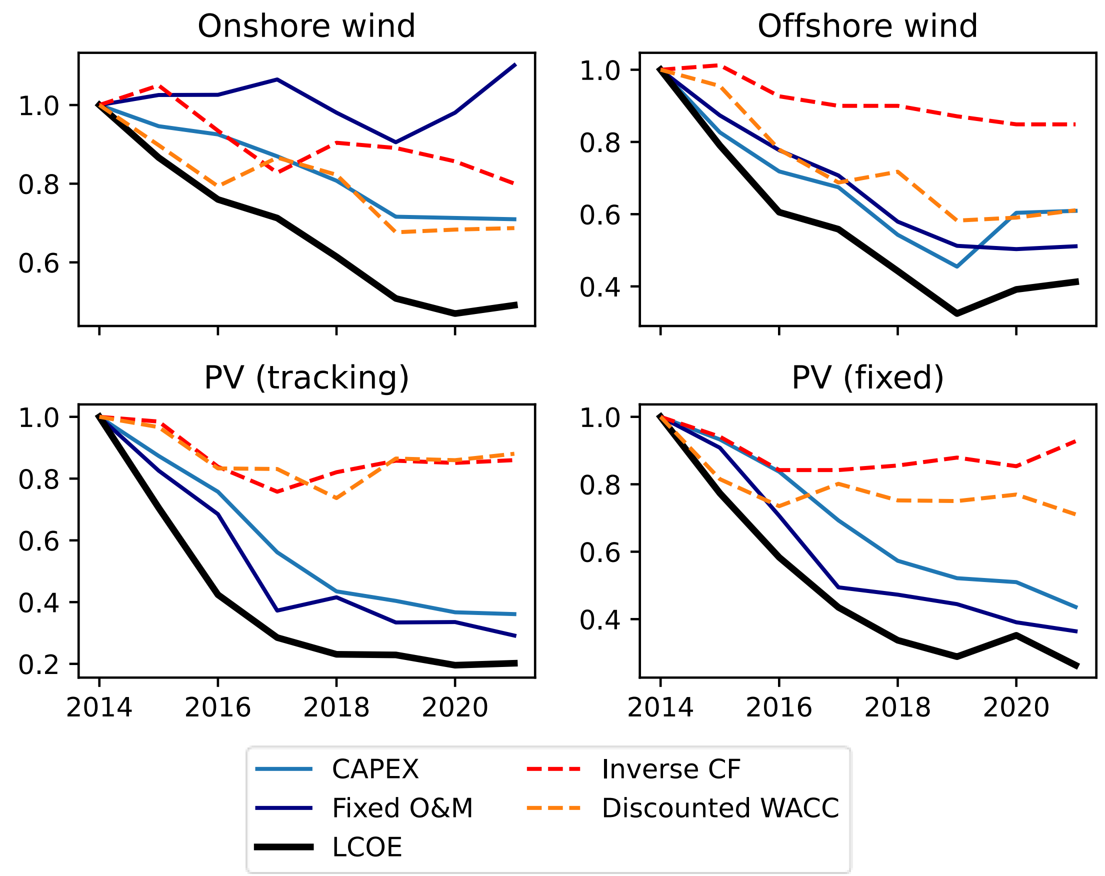

Supplementary Figure 4 ***Contributions to cost reductions of a selection of renewable technologies between 2014 and 2021****. In addition to capital expenditure (CAPEX) and fixed Operation and Maintenance (Fixed O&M), the inverse of the capacity factor (CF) is shown as well as the discounted Weighted Average Cost of Capital (WACC). Data derived from Bloomberg new Energy Finance.*

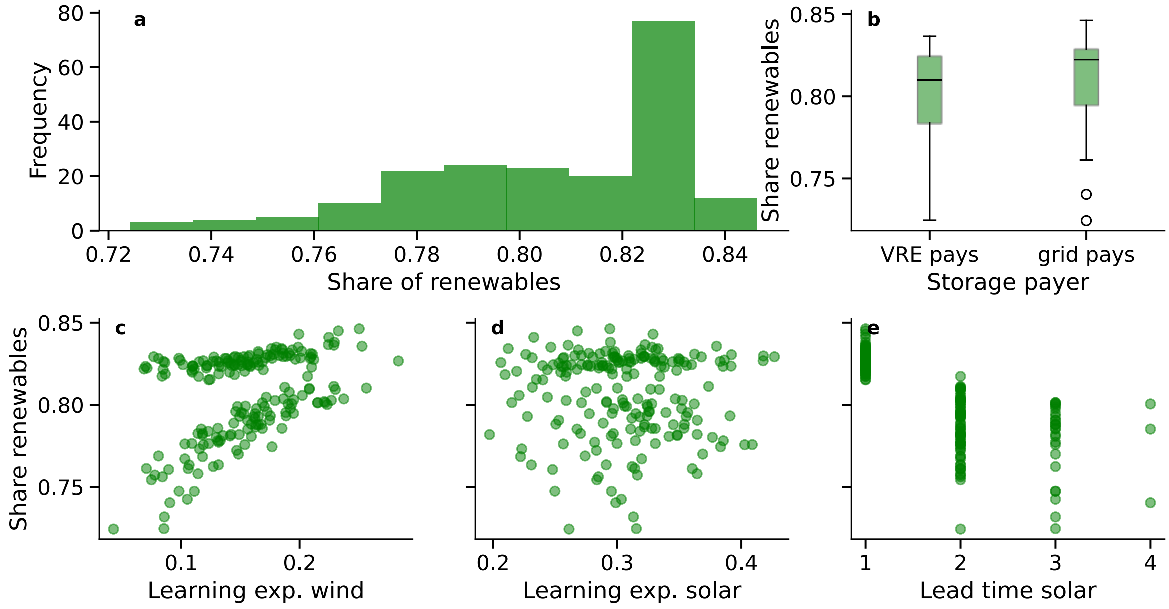

Supplementary Figure 5: Share of total renewables (wind + hydro + solar + geothermal) energy depending on key inputs, similar to Figure 5 of the main manuscript . ***a,*** *The overall histogram of the 2050 shares of renewables.* ***b,*** *The shares of*

renewables depending on who pays for storage costs (variable renewable energy (VRE) sources, or the grid operator). Box plot elements: Centre line: median, box limit: upper and lower quartiles, whiskers: 1.5x interquartile range, points: outliers ***c,*** *Shares of renewables depending on the learning rate of onshore and offshore wind energy,* ***d,*** *depending on the learning rate of solar PV and* ***e,*** *depending on the lead time for solar projects.*
